# Effect of Microstructure on Oxidation Resistance and TGO Formation in FeCoNiCrAl HEA Coatings Deposited by Low-Temperature HVAF Spraying

**DOI:** 10.3390/ma18071569

**Published:** 2025-03-30

**Authors:** Hossein Shahbazi, Rogerio S. Lima, Pantcho Stoyanov, Christian Moreau

**Affiliations:** 1 Department of Mechanical, Industrial and Aerospace Engineering, Concordia University, Montreal, QC H3G 1M8, Canada; 2 National Research Council of Canada, 75 de Mortagne Blvd, Boucherville, QC J4B 6Y4, Canada; rogerio.lima@cnrc-nrc.gc.ca; 3 Department of Chemical and Materials Engineering, Concordia University, Montreal, QC H3G 1M8, Canada; pantcho.stoyanov@concordia.ca

**Keywords:** thermal spray, thermal barrier coatings (TBCs), high entropy alloys (HEAs), bond coats, HVAF-i7

## Abstract

The effects of microstructure, density, and porosity of a FeCoNiCrAl high-entropy alloy (HEA) coating, fabricated using an internal diameter high-velocity air fuel (ID-HVAF) torch (model: i7 ID), on the isothermal oxidation behavior were investigated. This study pioneers the use of the ID-HVAF i7 ID system for HEA bond coat manufacturing, achieving a highly dense microstructure because of its low-operating spray temperature technique. To elucidate these effects, the microstructure and chemistry of the coating, the growth of the thermally grown oxides (TGOs), the phase transformation of alumina, and the oxidation rate were investigated at different temperatures. After 50 h at 1000 °C, 1100 °C, and 1150 °C, a dense, uniform, and thin alumina TGO layer (1.8 μm) was observed. The results demonstrate that the oxidation resistance of the HEA coating is enhanced because of the dense microstructure achieved via HVAF-i7, characterized by low porosity and uniform phase distribution, which contribute to improved barrier properties against oxygen diffusion. The growth of the TGO layer is controlled, resulting in a dense and continuous TGO layer. However, with increasing temperature and time, the alumina TGO layer becomes spalled, which is attributed to the absence of reactive elements. Overall, this study reveals that the FeCoNiCrAl HEA exhibits significant potential for enhancing oxidation resistance at high temperatures.

## 1. Introduction

Thermal barrier coatings (TBCs) play a critical role in modern gas turbines, aerospace engines, and other high-temperature applications by protecting essential components from thermal degradation and extending their operational lifespan. A key element of TBCs is the bond coat, which serves as an intermediate layer that enhances adhesion between the substrate and the thermal barrier top coat. Additionally, the bond coat provides essential resistance to oxidation and corrosion, ensuring durability in demanding operating conditions [1,2]. While traditional bond coat materials, such as MCrAlX alloys, where M represents Ni, Co, or NiCo, and X includes elements such as Y, Hf, Ta, and Si, have shown satisfactory performance, ongoing research continues to explore alternative materials with superior properties to enhance the durability and reliability of TBC systems further [3,4,5,6]. Among the promising candidates, FeCoNiCrAl high-entropy alloys (HEAs) stand out because of their exceptional mechanical properties and outstanding oxidation resistance at elevated temperatures [1,7,8,9,10,11,12,13,14,15,16,17,18,19,20,21,22,23,24]. In the realm of thermal spray techniques employed for applying bond coats, several methods have been developed, each offering unique advantages and challenges [1,8,25,26,27,28,29,30,31]. Notably, high-velocity air fuel (HVAF) stands out for its ability to produce coatings with dense microstructures, which is a very desired characteristic for improving TBC performance [21,32,33,34,35,36,37] by reducing coating porosity, unlike conventional techniques such as high-velocity oxy-fuel (HVOF), HVAF utilizes air instead of oxygen as the combustion medium, resulting in lower particle temperatures and higher particle velocities during the spraying process [21]. This advanced approach effectively minimizes in-flight oxidation and promotes the formation of high-quality, dense coatings with low as-sprayed oxidation levels. Among HVAF systems, the ID-HVAF i7 torch enables the deposition of coatings within internal diameters as small as 120 mm while maintaining precise control over coating quality and porosity levels [1]. A critical factor influencing HVAF performance is the selection of an appropriate particle size distribution (PSD), which plays a key role in determining the quality of the resulting microstructure. In their study, Shahbazi et al. investigated the HEA bond coat deposited using the HVAF process with an M3 gun, identifying an optimal PSD of 15–45 μm for achieving high-quality coatings [24]. Additionally, they examined the same FeCoNiCrAl HEA composition with a 15–45 μm PSD using the HVAF-i7 gun to assess the impact of a lower-temperature spray system. While this approach resulted in a dense and uniform bond coat, it did not achieve significant coating thickness. Based on the findings in the literature and the experimental experience of our team, a PSD of 5–30 μm was identified as optimal for the HVAF-i7 gun and was thus selected for this study [1,2]. Mauer et al. investigated the potential of the HVAF-M3 system for producing bond coats in TBC applications, focusing on conventional MCrAlY materials [38]. Their findings highlighted several advantages of HVAF over traditional methods. For example, atmospheric plasma spraying (APS) operates at high temperatures, leading to oxide formation during and after spraying, while HVOF uses oxygen in its fuel system, further promoting oxidation and increasing operational costs. As a result, HVAF emerges as a more cost-effective and efficient alternative. Additionally, the study compared the performance of two HVAF nozzles, 4L2 and 4L4, revealing a slight increase in deposition efficiency with higher particle velocities. The 4L4 nozzle achieved the maximum particle velocity at a spray distance of 350 mm, approximately 50 mm farther downstream than the optimal 300 mm spray distance for the 4L2 nozzle.

This study explores the potential of FeCoNiCrAl HEA as a replacement for conventional bond coats in TBC systems. By leveraging the unique properties of FeCoNiCrAl HEA and the advanced capabilities of HVAF technology, the goal is to develop dense microstructures that minimize spallation and facilitate the formation of a continuous, slow-growing thermally grown oxide (TGO) layer. To achieve this, the isothermal oxidation behavior of FeCoNiCrAl HEA coatings is systematically examined at temperatures ranging from 1000 °C to 1150 °C for 50 h. This investigation provides comprehensive insights into the TGO layer’s evolution under high-temperature conditions, evaluating the feasibility of FeCoNiCrAl HEA for enhancing TBC performance in demanding applications.

## 2. Materials and Methods

This study investigated the effects of microstructure, density, and porosity of FeCoNiCrAl HEA coatings fabricated using the ID-HVAF i7 torch (UniqueCoat Technologies, Oilville, VA, USA) on the isothermal oxidation behavior. To the best of the authors’ knowledge, this is the first study to employ the i7 ID torch to spray a FeCoNiCrAl HEA bond coat for TBC applications. The research examined the coating’s microstructure and chemistry, TGO growth, alumina phase transformation, and oxidation rate at different temperatures.

**Figure 1:**
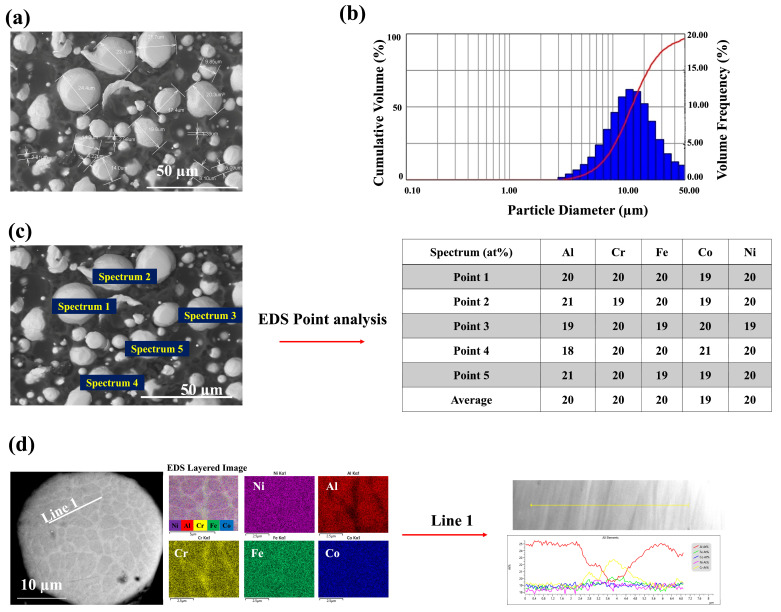
Characterization of FeCoNiCrAl HEA powder: (**a**) SEM image showing particle morphology and size measurements; (**b**) Particle size distribution measured using laser diffraction; (**c**) SEM-EDS image with marked spectrum points and corresponding elemental composition (at%) from point analysis; (**d**) Cross-sectional SEM image of a particle showing EDS elemental mapping (Al, Cr, Fe, Co, Ni) and EDS line scan across Line 1.

The characterization of the powder included particle size distribution analysis using a wet laser diffraction particle size analyzer (Spraytec, Malvern Panalytical Ltd., Malvern, UK) and cross-sectional powder morphology analysis via scanning electron microscopy (SEM) (S-3400N, Hitachi High Technologies America, Inc., Tokyo, Japan), equipped with a photodiode BSE detector, as shown in Figure 1. The chemical composition was determined using energy-dispersive X-ray spectroscopy (EDS). The EDX line scan in Figure 1 confirms the presence of Cr-rich and Al-rich regions within individual HEA powder particles, indicating microsegregation due to the sluggish diffusion effect inherent in HEAs. This segregation leads to compositional variations within the particles yet prevents significant phase separation after spraying, contributing to a more stable and uniform microstructure.

Before applying the bond coat, the substrates underwent grit blasting with Al_2_O_3_ grit #80 at 4.1 bar (60 psi) to increase surface roughness and enhance coating adhesion. The substrate’s roughness plays a critical role in how thermal spray particles interact with the surface during deposition.

In HVAF and HVOF processes, particles impacting a rough surface can deform and fill surface irregularities more effectively, contributing to a denser microstructure. However, if the surface is too smooth, particle adhesion may be insufficient, leading to a lower coating density. Conversely, an excessively rough surface may prevent particles from fully filling the valleys, potentially increasing coating porosity [29]. This process resulted in an average surface roughness (Ra) of 3.9 ± 0.6 μm on the Inconel 718 substrate. The Ra measurements were performed using a Mitutoyo-SJ.210 profilometer (Mitutoyo Corporation, Kawasaki, Japan) with a cut-off length of 2.5 mm, averaging ten measurements.

Isothermal oxidation tests were conducted on puck-shaped Inconel 718 substrates with a 25.4 mm diameter and 6 mm thickness. The ID-HVAF i7 system employs large converging-diverging nozzles and long, large-diameter powder injectors for both preheating and spraying, using propylene as fuel. The extended nozzle increases particle residence time in the hot combustion gas, promoting a dense microstructure. This torch produces high-quality coatings with porosity levels below 1%.

To prevent clogging issues associated with smaller injectors, the 3L4 nozzle (long nozzle: 63 mm) was paired with a large-diameter powder injector (2.75 mm). The HVAF parameters used with the i7 gun are summarized in Table 1.

During the spraying process, a ThermoVision A320 infrared camera (FLIR Systems, Inc., North Billerica, MA, USA) was used to monitor the substrate’s surface temperature, with a preheating temperature recorded at 155 °C. To preserve the actual microstructures, the as-sprayed bond coat samples were vacuum-impregnated in epoxy resin and hardener (ANAMET, Frankford, ON, Canada). The samples were then mounted, cut, and polished following standard metallographic procedures.

The cross-sectional microstructure of the bond coats was analyzed using a Field Emission Scanning Electron Microscope (FESEM, Hitachi, Regulus 8230, Tokyo, Japan). The elemental compositions of the FeCoNiCrAl HEA bond coat and the TGO layer were assessed using energy-dispersive X-ray spectroscopy (EDS) and elemental mapping techniques. For isothermal oxidation testing, a tubular furnace with a maximum operating temperature of 1200 °C (Barnstead Thermolyne, Model F21135, Dubuque, IA, USA) was used.

## 3. Results and Discussions

### 3.1. The Microstructure of the Coatings

**Figure 2:**
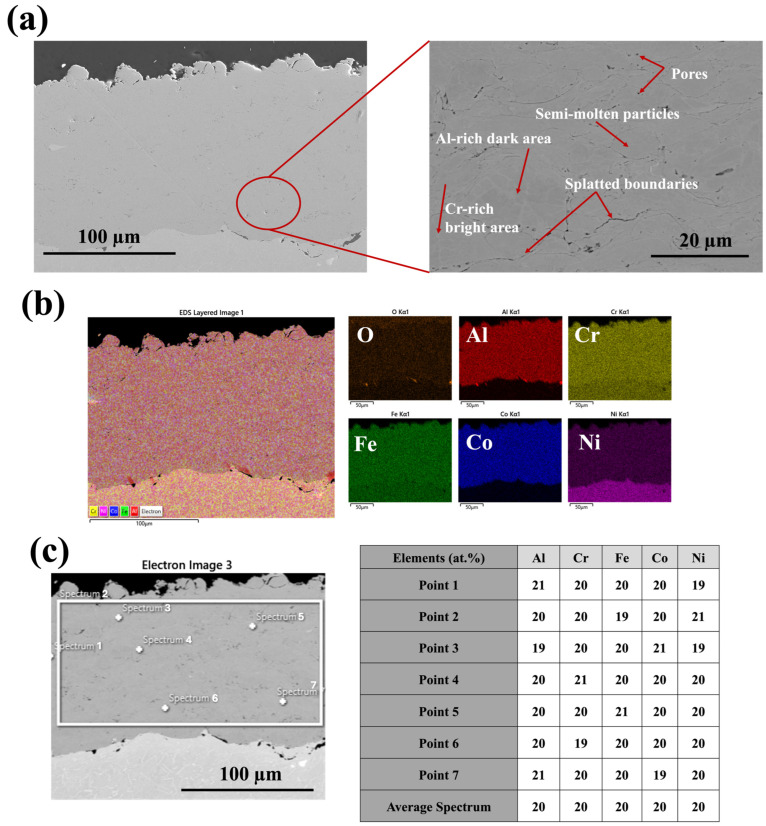
FESEM images of the as-sprayed microstructure of FeCoNiCrAl HEA using the HVAF i7 gun: (**a**) Cross-sectional view of the as-sprayed HEA bond coat, (**b**) Elemental mapping showing uniform distribution and random solid solution, (**c**) EDS area and point analysis (white rectangle indicating the EDS area).

Figure 2 shows the cross-sectional microstructure of the HEA bond coat sprayed via an HVAF-i7 torch with a long nozzle and large-diameter powder injectors. Based on our previous study, FeCoNiCrAl HEA was sprayed with different air and fuel pressure parameters [24]. The highest deposition efficiency and lowest porosity in the microstructure were achieved using medium air and fuel pressure, as detailed in Table 1. The deposition efficiencies (DEs) for low, medium, and high air and fuel pressures were 73%, 79%, and 53%, respectively, while the corresponding porosity levels were 0.8%, 0.2%, and 1.8%. Porosity measurements were conducted using ImageJ (Version 1.53t) analysis.

As mentioned earlier, the porosity of HVAF-i7 coatings typically falls around 1%. However, the use of propylene as a fuel—due to its higher calorific value and ability to generate a narrow, stable flame—combined with optimized spray parameters enabled the achievement of a significantly lower porosity of 0.2% and a dense microstructure. This dense structure minimizes oxygen ingress, thereby substantially reducing internal oxidation in the bond coat compared with coatings with higher porosity (>6%). While it does not directly mitigate TGO thickening, the reduction in internal oxidation helps maintain the bond coat’s integrity and delays spallation at high temperatures [39]. Examining the microstructure reveals that the intermediate inter-diffusion zone (IDZ) at the coating/substrate interface is approximately 3 μm thick, indicating a similar level of inter-diffusion throughout. Due to the relatively thin inter-diffusion layer (~3 μm) compared with the thicker coating (~150 μm), the impact of inter-diffusion on the oxidation behavior can be considered negligible [40]. As shown in Figure 2, the cross-sectional low-magnification image reveals a fully dense microstructure, demonstrating the effectiveness of the HVAF-i7 process in producing high-density coatings because of its low-temperature operation. At higher magnification, some semi-molten areas are visible. These features result from the high-velocity impact of particles onto the substrate, where kinetic energy facilitates coating densification. In some cases, the temperature generated by particle collisions may lead to the presence of semi-molten or unmolten regions within the microstructure.

In Figure 1, two distinct areas—dark and bright—are visible, corresponding to Al-rich and Cr-rich regions, respectively. This suggests microsegregation within the cross-sectional powder. Light elements such as Al appear as dark areas in the microstructure, while heavy elements such as Cr stand out as bright areas. Therefore, it is advisable not to categorically identify these areas as distinct phases without significant deviations in the EDS line analysis fluctuation graphs. Instead, it indicates that random solid solution phases were achieved using the HVAF technique for the FeCoNiCrAl HEA composition. The advantage of using HEA as a bond coat is the absence of the β-phase (NiAl-rich phase), which is often found in conventional bond coats and can cause brittleness at lower temperatures [24]. As temperatures exceed the ductile-to-brittle transition temperature (DBTT), around 750 °C for NiAl, this brittleness reduces, allowing the material to endure more strain before fracturing [1]. Moreover, the β-NiAl phase, which typically appears as granules, is less apparent or missing near defects such as splat boundaries, voids, oxide stringers, and unmelted particles in APS and HVAF bond coats. The disappearance of the β-NiAl phase near these defects suggests that they negatively impact the phase’s integrity or consistency. Generally, the β-NiAl phase is valued for its high-temperature oxidation resistance and mechanical properties. However, its presence has both advantages and disadvantages, as discussed earlier [41]. By using HEA, the issues associated with the β-NiAl phase are avoided, resulting in improved oxidation resistance. This benefit will be further detailed in the subsequent section of this study.

**Figure 3:**
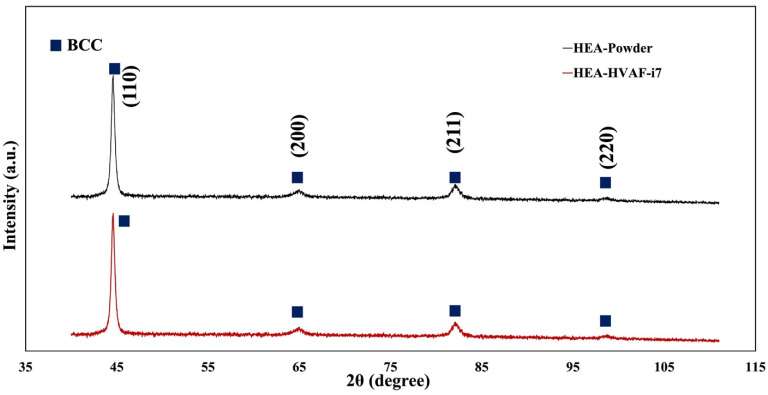
XRD patterns of FeCoNiCrAl HEA powder and HVAF-sprayed coating.

Figure 3 shows the XRD patterns of the HEA powder and the HVAF-i7 deposited coating, indicating that no significant phase transformations occurred during the spraying process. Both conditions exhibit a body-centered cubic (BCC) structure, confirming that the high-velocity spraying process effectively preserves the original phase composition and promotes the formation of a random solid solution.

Moreover, pseudo-Voigt fitting was performed on the four main diffraction peaks to analyze the peak profiles quantitatively. The extracted FWHM values for the powder and the coating are provided in Table 2. The results reveal that the FWHM values of the coating are nearly identical to those of the feedstock powder, indicating that no considerable crystallite refinement or strain evolution occurred during deposition. This observation suggests that the HVAF-i7 process preserves not only the phase composition but also the crystallite characteristics of the initial powder. Consequently, the process produces a dense and phase-stable coating with minimal microstructural changes, reinforcing its potential as a suitable HEA bond coat for thermal barrier coating applications.

Furthermore, as shown in Figure 2, EDS mapping and area analysis confirm the formation of a random solid solution phase with a uniform elemental distribution after coating. This highlights the effectiveness of the HVAF technique and the intrinsic high-entropy effect, which promotes the development of a solid solution phase within a simple crystalline structure [1,42].

As previously mentioned, roughness plays a critical role in determining coating quality in TBCs. Both substrate and bond coat roughness are essential for enhancing adhesion, increasing bonding strength, and reducing stress at the bond coat–top coat interface. A rougher bond coat surface promotes mechanical interlocking with the ceramic top coat, improving adhesion and minimizing the risk of delamination [29]. Increased roughness also enhances the surface area available for top coat adhesion, further improving bonding strength. A rough bond coat surface helps distribute thermal stress more evenly, reducing localized stress concentrations that could lead to cracking and spallation. Controlled roughness promotes the formation of a uniform and well-adhered thermally grown oxide (TGO) layer, which is essential for oxidation resistance. However, excessive roughness may introduce stress concentrations, increasing the risk of premature failure [43].

Optimal roughness enhances thermal barrier properties by strengthening the bond between layers and improving the system’s overall thermal insulation efficiency. Enhanced adhesion due to optimal roughness also increases fatigue resistance, particularly under cyclic thermal and mechanical loads, such as those experienced in gas turbines and aeroengines. Achieving the ideal roughness through techniques such as grit blasting is crucial for ensuring reliable TBC performance in high-temperature applications. Generally, an average roughness (Ra) of 3 to 5 μm provides a balance between mechanical interlocking and maintaining a smooth surface to prevent stress concentrations. In this study, coating roughness was measured using a Mitutoyo-SJ.210 profilometer with a 2.5 mm cut-off length, averaged over ten measurements, yielding an Ra value of 4.7 μm.

### 3.2. TGO Microstructure and Growth Kinetics

Prolonged exposure to high temperatures (exceeding 100 h) resulted in unsatisfactory outcomes, likely because of the melting point limitations of the Inconel 718 substrate. This led to partial substrate melting, which compromised coating integrity. To address this issue, Inconel 625 has been selected for future studies, with further investigations planned to evaluate its performance under similar conditions.

Gupta et al. explored strategies to enhance the lifespan of SPS thermal barrier coatings, focusing on bond coats deposited using the HVAF process with the M3 supersonic gun [44]. Isothermal oxidation tests were conducted at 1100 °C for 10, 50, 100, and 200 h. Their results revealed the formation of a uniform, dense, and continuous alumina TGO layer after 50 h of exposure. Additionally, a mixed oxide TGO began to develop in the MCrAlY bond coat as the test progressed. Notably, even after 200 h of oxidation, the TGO thickness remained well-controlled at approximately 6 μm.

In contrast, the present study observed no formation of mixed oxides after 50 h at 1150 °C. This can likely be attributed to two key factors: (i) the sluggish diffusion characteristics inherent to HEAs, which reduce the transport of elements such as Cr and Al, thereby limiting the formation of complex oxides, and (ii) the use of the HVAF-i7 gun, a lower-temperature spraying technique that minimizes oxidation during deposition. However, a thermally grown oxide (TGO), primarily Al_2_O_3_, was observed on the surface of the HEA bond coat over time, indicating that oxidation occurred but remained largely limited to the formation of a protective alumina scale rather than mixed oxide phases. The HVAF-i7 gun operates at reduced particle temperatures compared with the HVAF-M3, minimizing the formation of oxides during spraying and promoting the development of a more uniform, oxide-free microstructure. This reduced oxidation at the deposition stage further delays the formation of mixed oxides during subsequent high-temperature exposure [24]. After 50 h at 1000 °C, 1100 °C, and 1150 °C, a dense, uniform, and thin alumina TGO layer was observed in the HEA bond coat. These results suggest that the enhanced oxidation resistance of the HEA coating is likely attributable to the dense microstructure achieved using the HVAF-i7 process. While a direct comparison with benchmark bond coat systems (e.g., MCrAlY) is not available within this study, evidence from the literature indicates that the HVAF-M3 gun, which operates at higher particle temperatures, tends to introduce more oxides during deposition. By contrast, the lower operating temperature of the HVAF-i7 gun minimizes oxide formation during spraying, thereby promoting a more uniform and oxide-free bond coat microstructure. Additionally, the inherent sluggish diffusion characteristics of HEAs further suppress the transport of alloying elements such as Cr and Al, which are critical for forming protective oxides, thereby delaying the formation of mixed oxides during high-temperature exposure. Collectively, these factors likely contribute to the observed differences in TGO characteristics and highlight the potential advantages of combining HEAs with the HVAF-i7 s1praying technique.

The growth of the TGO layer remained controlled, resulting in a dense and continuous alumina TGO layer. In conventional bond coats, alumina TGO layers often spall at higher temperatures when reactive elements are absent. However, the FeCoNiCrAl HEA bond coat demonstrated enhanced oxidation resistance by promoting the formation of a uniform and adherent alumina TGO layer. This behavior may be attributed to the unique properties of HEAs, particularly sluggish diffusion, which can slow the movement of elements, thereby controlling TGO growth and reducing spallation risk, as observed in this study.

To monitor oxidation behavior, specimens were placed in alumina ceramic crucibles to collect any detached oxide scales during testing. After each specified exposure time, samples were removed from the furnace, air-cooled to room temperature for approximately 5 min, and reweighed while still in their respective crucibles. Each sample was weighed five times, with precautions taken to prevent oxide and ash weight loss. The weight gain due to TGO formation was recorded at each time interval, and the total weight difference (mg) for each sample was divided by the total surface area (coating + substrate) [cm^2^] to determine the specific weight gain [26,29,45,46,47]. This process ensured accurate measurement of the oxidation behavior and the development of the TGO layer over various durations and temperatures.

**Figure 4:**
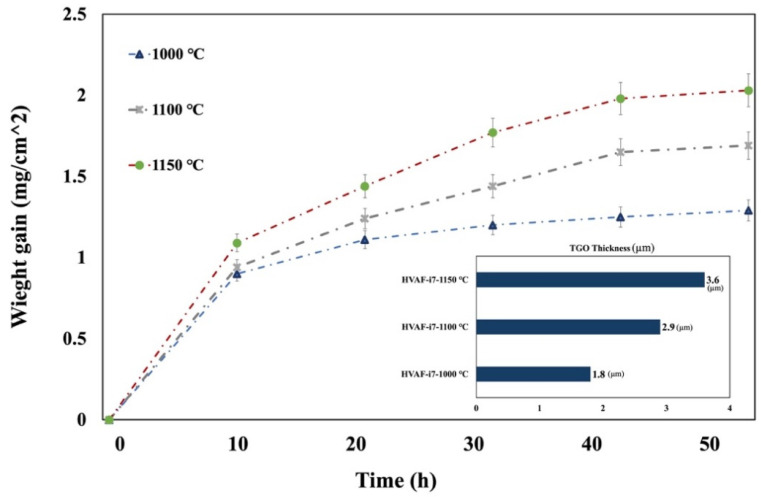
Isothermal oxidation test of the HEA bond coat applied using the HVAF i7 gun at different temperatures after 50 h. Error bars indicate the standard deviation.

The results of the isothermal oxidation tests at 1000, 1100, and 1150 °C after 50 h are shown in Figure 4. No visible spallation was observed in any sample, indicating that the oxide scales remained intact. These plotted data represent the oxidation rate as an increase in mass per unit area. The coatings deposited using the HVAF-i7 gun with HEA exhibit low thickness, attributed to the dense microstructure achieved through this process.

**Figure 5:**
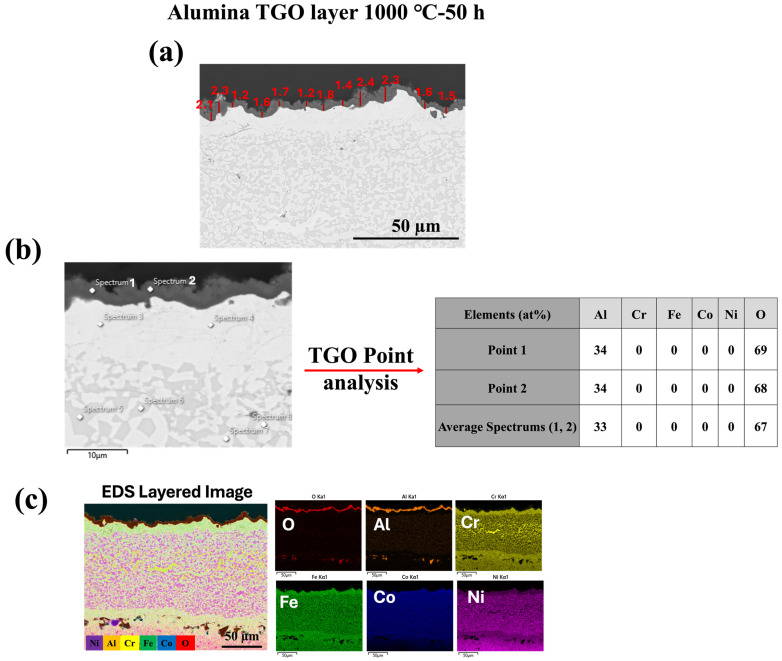
Characterization of TGO layer formed at 1000 °C for 50 h: (**a**) SEM cross-sectional image of the TGO layer with measured thickness values (in μm); (**b**) SEM image showing EDS point analysis locations, with the corresponding elemental composition (at%) of selected points within the TGO; (**c**) EDS layered image and elemental maps of the oxidized surface showing distribution of O, Al, Cr, Fe, Co, and Ni.

**Figure 6:**
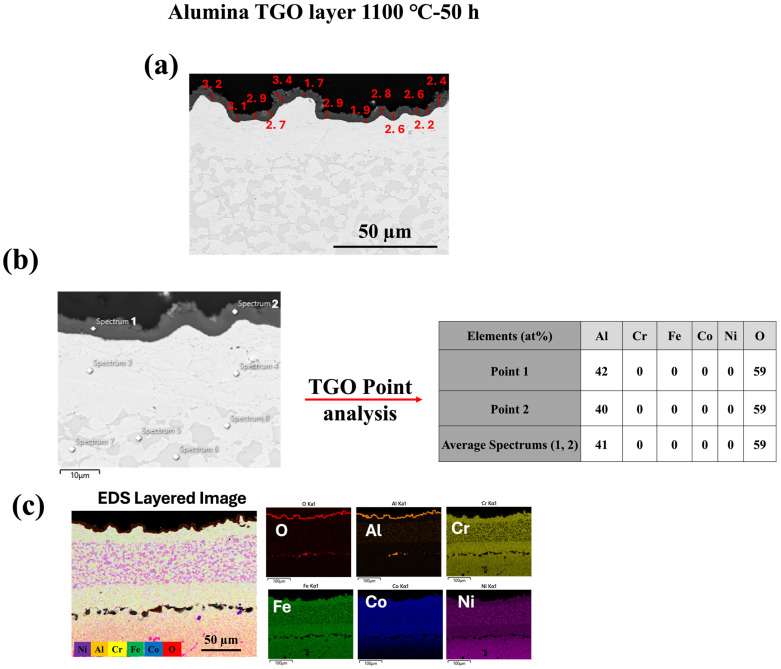
Characterization of thermally grown oxide (TGO) layer formed at 1100 °C for 50 h: (**a**) SEM cross-sectional image of the TGO layer with thickness measurements (in μm); (**b**) SEM image showing EDS point analysis locations with corresponding elemental compositions (at%) of selected points within the TGO; (**c**) EDS layered image and individual elemental maps showing the distribution of O, Al, Cr, Fe, Co, and Ni in the oxidized region.

**Figure 7:**
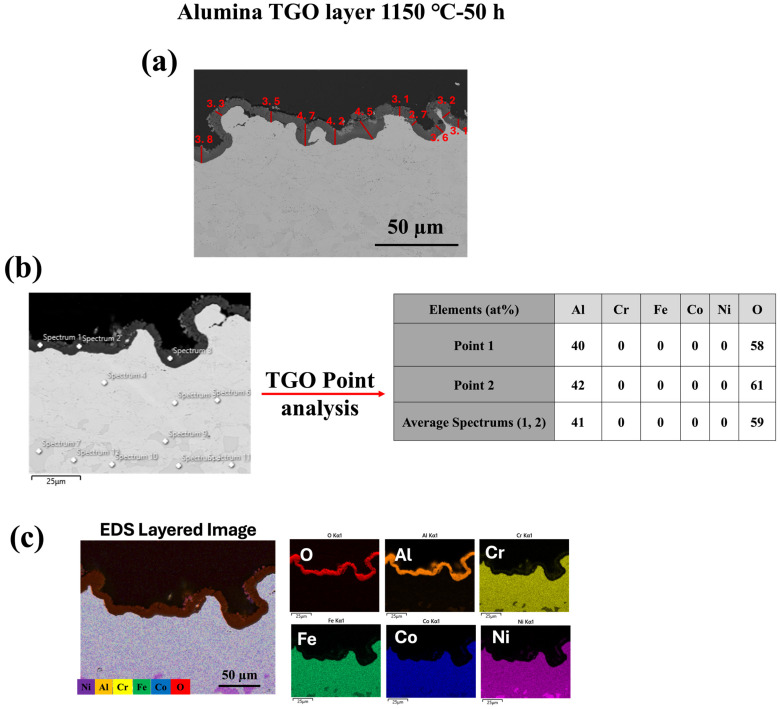
Characterization of thermally grown oxide (TGO) layer formed at 1150 °C for 50 h: (**a**) SEM cross-sectional image of the TGO layer with thickness measurements (in μm); (**b**) SEM image showing EDS point analysis locations with corresponding elemental compositions (at%) of selected points within the TGO; (**c**) EDS layered image and individual elemental maps showing the distribution of O, Al, Cr, Fe, Co, and Ni in the oxidized region.

As illustrated in Figure 5, Figure 6 and Figure 7, oxide growth increases with temperature at 1000 °C, 1100 °C, and 1150 °C, respectively. The HVAF-i7 technique, combined with HEA, effectively controls the growth of the TGO layer. Future research in our group will explore the incorporation of reactive elements such as Y and Hf to further regulate TGO growth.

The Al_2_O_3_ TGO layer formed uniformly and consistently across all isothermal oxidation tests, demonstrating the effectiveness of the HVAF-i7 process in conjunction with the FeCoNiCrAl HEA composition. No Al_2_O_3_ oxide scale spallation was detected. The TGO layer formation results from the oxidation reaction between oxygen diffusing through the top coat and aluminum ions in the bond coat. EDS point analysis confirms that in the diffusion zone, the aluminum content is lower than that of other elements, indicating that aluminum ions migrate from the bond coat to the top layer, contributing to TGO layer formation.

Shahbazi et al. investigated FeCoNiCrAl HEA coatings deposited using HVAF M3 and HVAF-i7 guns. Their study utilized a particle size distribution (PSD) of 15–45 μm, which was suitable for the M3 gun because of its higher operating temperature, similar to HVOF. However, this PSD was not ideal for the HVAF-i7 gun, which operates at a lower temperature and requires finer particles (5–30 μm). As a result, they were unable to achieve significant coating thickness or high deposition efficiency with HVAF-i7 [1].

The TGO layer formed at 1000 °C after 50 h measured 1.06 μm, but its accuracy was affected by the thin bond coat thickness (~20 μm). In contrast, this study used finer particles (5–30 μm), allowing the coating thickness to reach 150 μm, which is within the standard range for TBC systems (150–200 μm). The resulting TGO layer was thin, dense, continuous, and uniform, demonstrating excellent high-temperature performance. This improvement is attributed to the sluggish diffusion properties of HEAs, which regulate elemental diffusion at high temperatures. The unique atomic configuration of HEAs creates local energy variations, trapping atoms in low-energy regions and making the slowest-diffusing element the rate-determining factor for oxidation resistance [1,10,13,16,17].

**Figure 8:**
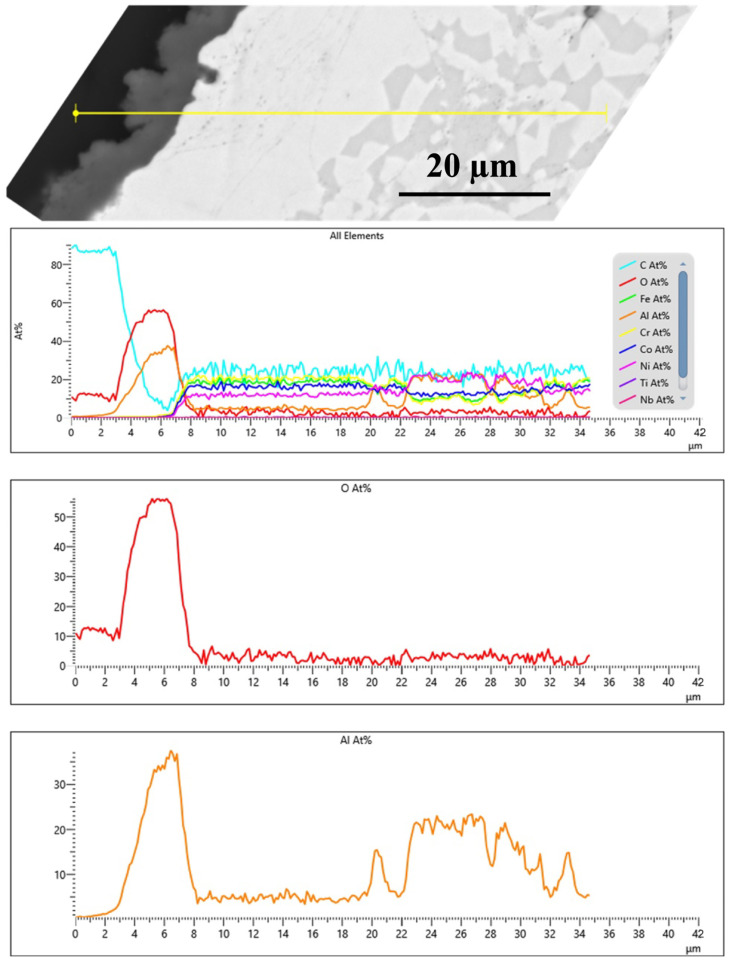
EDS line analysis of the cross-sectional microstructure (HVAF-i7) of the bond coat/TGO interface after 50 h of oxidation at 1000 °C.

Figure 8 presents the cross-sectional microstructure and EDX line analysis of the FeCoNiCrAl/TGO interface after 50 h of oxidation at 1000 °C, offering a detailed view of elemental diffusion and TGO formation, complementing the observations in Figure 5, Figure 6 and Figure 7. In Figure 5, Figure 6 and Figure 7 the formation of a dense alumina (Al_2_O_3_) TGO layer at the bond coat interface is evident, establishing the basis for analyzing the inter-diffusion zone. Figure 8 builds upon this by highlighting elemental migration as a key factor in TGO growth.

The EDX profiles reveal a sharp increase in oxygen (O) concentration at the TGO interface, confirming the presence of a protective alumina scale, as observed in Figure 5, Figure 6 and Figure 7. The aluminum (Al) profile shows noticeable fluctuations, with a significant drop at the oxide front, indicating outward diffusion from the bond coat to sustain the alumina layer. This depletion of Al in the bond coat, along with its accumulation at the oxide interface, reflects ongoing inter-diffusion, driving the growth of the alumina TGO layer [48,49]. The gradual decline and variability in the aluminum profile suggest regions of continuous TGO thickening, while the oxygen profile stabilizes past the TGO layer, reinforcing the barrier effect of alumina. These compositional trends validate that the alumina scale formed is dense, adherent, and resistant to further oxidation. The absence of significant chromia or spinel oxide peaks, as previously observed in Figure 5, Figure 6 and Figure 7, underscores the superior oxidation resistance of alumina-based TGO compared with mixed oxide layers. This reinforces the role of FeCoNiCrAl coatings in promoting long-term TBC performance by limiting oxygen diffusion, reducing spallation risks, and ensuring enhanced stability under high-temperature exposure [28,50].

## 4. Conclusions

This study investigates the influence of microstructure, density, and porosity of fine FeCoNiCrAl HEA coatings, manufactured through HVAF-i7, on their isothermal oxidation behavior.

(1) Utilizing the HVAF-i7 gun, we achieved a highly dense microstructure comparable to that of cold spray techniques, highlighting the efficacy of the fabrication method.
(2) The microstructure and chemistry of the coating, along with the growth of the thermally grown oxide (TGO) layer, were investigated across different temperatures, revealing a dense, uniform, and thin alumina TGO layer after 50 h at 1000 °C, 1100 °C, and 1150 °C. It was observed that with increasing temperature, the TGO layer increased, albeit not significantly.
(3) The oxidation resistance of the HEA coating was significantly enhanced by the dense microstructure achieved via HVAF-i7, which contributed to the controlled growth of the TGO layer and the formation of a continuous alumina scale. While no direct benchmark bond coat was included for comparison, the observed microstructural characteristics and TGO behavior align with the known performance benefits associated with dense coatings produced through HVAF processes.
(4) The absence of reactive elements contributed to the spalling of the alumina TGO layer at higher temperatures, which will be further explored in future research.
(5) Analysis of deposit efficiency (79%) and porosity (0.2%) indicated that the best deposition efficiency and lowest porosity were achieved with medium air and fuel pressure, resulting in a dense, uniform, and random solid solution phase.
(6) Investigation of the microstructure revealed a relatively thin inter-diffusion layer compared with the coating thickness, suggesting a negligible impact on oxidation behavior.
(7) EDS mapping and area analysis confirmed the presence of a random solid solution phase and uniform element distribution after coating, highlighting the efficacy of the HVAF technique and the high entropy properties.

## Figures and Tables

**Table 1:** Spray parameters for bond coat production using the HVAF i7 ID torch.

Parameters	Value
Fuel pressure (bar)	7.72
Air pressure (bar) (P1/P2)	8.96
Carrier gas flow rate (L/min)	25
Powder feed rate (g/min)	120
No. of passes	5
Preheat (passes)	5
Torch traverse speed (mm/s)	1000
Spray distance (mm)	50.8
Substrate temperature (FLIR systems)	155 °C

**Table 2:** FWHM values of the four major peaks for the powder and HVAF-i7 coating obtained by pseudo-Voigt fitting.

Peak (hkl)	FWHM (Powder) (°)	FWHM (HVAF-i7 Coating) (°)	Difference (°)
(110)	0.46124	0.46131	0.00007
(200)	1.123	1.123	0.00000
(211)	0.882	0.896	0.01400
(220)	1.684	1.703	0.01900

## Data Availability

The original contributions presented in this study are included in the article. Further inquiries can be directed to the corresponding authors.
